# Pilot Evaluation of a New Urine Progesterone Test to Confirm Ovulation in Women Using a Fertility Monitor

**DOI:** 10.3389/fpubh.2019.00184

**Published:** 2019-07-02

**Authors:** Thomas P. Bouchard, Richard J. Fehring, Mary Schneider

**Affiliations:** ^1^Department of Family Medicine, University of Calgary, Calgary, AB, Canada; ^2^College of Nursing, Marquette University, Milwaukee, WI, United States

**Keywords:** natural family planning, progesterone, fertility monitoring, ovulation, ovulation confirmation

## Abstract

**Background:** Progesterone rises ~24–36 h after ovulation. Past studies using ultrasound-confirmed ovulation have shown that three consecutive tests with a threshold of 5μg/mL of urine progesterone (pregnanediol-3-glucuronide, PDG), taken after the luteinizing hormone (LH) surge, confirmed ovulation with 100% specificity.

**Purpose:** The purpose of this study was to a evaluate a new urine PDG self-test to retrospectively confirm ovulation in women who were monitoring ovulation using a hormonal fertility monitor.

**Methods:** Thirteen women of reproductive age were recruited to test urine PDG while using their home hormonal fertility monitor. The monitor measured the rise in estrogen (estrone-3-glucuronide, E3G) and LH to estimate the fertile phase of the menstrual cycle. The women used an online menstrual cycle charting system to track E3G, LH and PDG levels for four menstrual cycles.

**Results:** The participants (*Mean* age 33.6) produced 34 menstrual cycles of data (*Mean* length 28.4 days), 17 of which used a PDG test with a threshold of 7μg/mL and 17 with a threshold of 5μg/mL. In the cycles that used the 7μg/mL test strips, 59% had a positive confirmation of ovulation, and with the 5μg/mL test strips, 82% of them had a positive confirmation of ovulation.

**Conclusion:** The 5μg/mL PDG test confirmed ovulation in 82% of cycles and could assist women in the evaluation of the luteal progesterone rise of their menstrual cycle.

## Introduction

Teaching women to monitor their menstrual cycle can empower them to understand their reproductive health in order to facilitate or avoid pregnancy (1–3). Women have been able to monitor estrone-3-glucuronide (E3G) and luteinizing hormone (LH) at home in their urine using test sticks and fertility monitors for several decades, but monitoring urine pregnanediol-3-glucuronide (PDG, the urine metabolite of progesterone) has been very limited. The most common way to assess the luteal phase and confirm ovulation has been to take daily first morning basal body temperatures to track a significant rise from baseline. This temperature method is time consuming and often inaccurate (4). Confirming ovulation with a serum progesterone test (5) requires a physician order and going to the laboratory to draw blood, which is expensive and time consuming. A fertility monitor measuring PDG has been developed since the 1980s by Brown and colleagues (6) but these devices are currently not widely available (7).

A new urine PDG test (Progesterone Ovulation Test “Proov,” https://proovtest.com) is now commercially available and is currently being used by women, including users of the Marquette Method, to evaluate their progesterone status. The Marquette Method is a Fertility Awareness Based Method (FABM) that uses the ClearBlue Easy Fertility Monitor (CBEFM) to monitor urinary hormones of the menstrual cycle with an online education and charting application (nfp.marquette.edu). The CBEFM measures the rise in E3G and the surge in LH to estimate the fertile phase of the menstrual cycle (8, 9). In order to understand and educate users on the utility and accuracy of the new PDG test stick, we undertook the current pilot study of the PDG test strip using a simple protocol. The protocol was tested by Marquette Method health professionals (i.e., physicians and professional nurses), providers and users.

The new PDG test strip indicates when a threshold level of progesterone has been reached after a Luteinizing Hormone (LH) surge and/or the peak of cervical mucus. The threshold level of progesterone detected by the PDG test strip was based upon a research study (10) that evaluated urinary levels of PDG after a luteinizing hormone (LH) surge or the peak of cervical mucus. It was found that ultrasound-confirmed ovulation could be identified retrospectively with 100% specificity when urinary PDG thresholds were more than 5μg/mL for three consecutive days following an LH surge or peak mucus (10).

The purpose of this pilot study was to test the use of the new urine PDG tests by comparing the new test's confirmation of ovulation with the CBEFM's detection of the LH surge. The developers of the ClearBlue monitor have demonstrated the strong correlation between ovulation and the LH surge (11), and in the present study, sensitivity of the PDG assay in confirming ovulation is based on the LH surge, which is a reliable surrogate for ovulation. The LH surge occurs 24–36 h before ovulation (12) and was used to determine the percentage of positive PDG tests that occurred after the LH surge. This was a feasibility pilot study of the use of the new PDG test strip and there is only one other study that we are aware of looking at the feasibility of the use of this test for women's cycles. Before we can recommend the use and accuracy of this test strip for Marquette Method users, we wanted to demonstrate a level of evidence in the field that the manufacturer has not yet produced.

## Methodology

Marquette Method providers and users who use an online menstrual cycle charting system volunteered for the study. The providers were sent an e-mail asking them to consider participating in a study to test the new PDG test strips. We planned to recruit initially only 10 participants, but 17 women agreed to participate and signed a consent form, 13 of whom contributed from 1 to 4 menstrual cycles of data. The volunteer women users or teachers of the Marquette Method were sent the PDG test strips (test strips for both 7 and 5μg/mL thresholds) and given instructions to use the strips for 1–4 menstrual cycles and to chart the results in the Marquette Method online charting system.

Inclusion criteria were: (1) regular menstrual cycles, (2) at least 3 menstrual cycles after cessation of breastfeeding, (3) age 20–46, (4) a Marquette Method provider or user using a Clearblue Easy Fertility Monitor (CBEFM), (5) willing to complete one to four menstrual cycles of testing, and (6) willing to provide us with completed menstrual cycle charts using the CBEFM and the urine PDG tests. Exclusion criteria were: (1) a history of polycystic ovarian syndrome or (2) sub-fertility. Participants were given 6 months free use of the online charting site.

**Figure 1:**
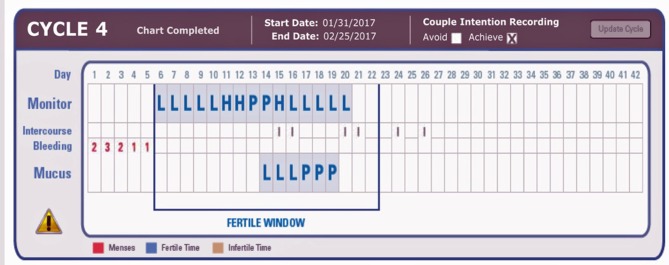
Example charts showing three positive PDG tests (on the mucus line) after the LH surge (P for Peak) detected by the ClearBlue Easy Fertility Monitor. L, negative test; P, positive test. On the Monitor Line, L, Low; H, High; P, Peak fertility based on E3G and LH results from the CBEFM.

The volunteer participants were asked to follow the following protocol which was approved through the Marquette University's Institutional Review Board (IRB):

1. On the morning of the second Peak LH reading of the monitor use the PDG test with your first morning urine collected and tested in a container (not within the urine stream).
2. Continue to use the PDG test with the first morning urine until you have three positive tests in a row (Note that three positive tests in a row is a confirmation of ovulation).
3. If you think you have a missed LH surge with the monitor do not use the PDG test that cycle; wait until the next cycle when you have a peak on the monitor.
4. Record the results on the bottom (“Mucus”) line of the online chart (Figure 1), and record a positive test as “P” and a negative test as “L.”

The use of the “Mucus” line on the online chart was to facilitate recording in the current online charting system without having to create an entirely new charting system for the PDG result (Figure 1). The indicators “P” and “L” refer to “Peak” and “Low,” respectively, and are used as descriptors for the Mucus rating scale in the ordinary use of the Marquette Method.

## Results

The 13 volunteer participants (*Mean* age 33.6; *SD* = 6.4; range 26–46) produced 34 menstrual cycles of data (*Mean* length 28.4 days; *SD* = 2.1; range = 23–32 days). The luteal phase of our participants ranged from 10 to 16 days.

There were 17 menstrual cycles of data using test sticks for each of the 7 and 5 μg/mL thresholds (Table 1). Only 59% (10 of 17) of the menstrual cycles that used the 7 μg/mL test strips had a positive PDG test while 82.4% (14 of 17) had a positive PDG test using the 5 μg/mL test strips (Table 1).

**Table 1:** Frequency of positive PDG tests (5 and 7 μg/mL) in days after the LH surge.

**Days Past LH Surge**	**5 ug/mL**	**7 ug/mL**
1	2	1
2	1	2
3	6	4
4	5	1
5	0	1
6	0	0
7	0	0
8	0	1
9	0	1
Total Positive Tests	14	11
Negative Tests	3	7
Total Tests	17	18
Percentage	82%	59%

The positive PDG tests occurred from 2 to 10 days after the second Peak reading of LH on the monitor, with the most frequent positive results on days 4 and 5 past the surge. However, all 14 of the menstrual cycles that had a positive PDG with the 5 μg/mL test strips had a narrower window, occurring from 2 to 5 days past the LH surge with the most frequent positive test on days 4 and 5 (see Table 1). Confidence intervals (95%) were 0.64 to 1.00 for the 5 μg/mL test and 0.36 to 0.82 for the 7μg/mL test.

## Discussion

We found that the urine PDG test strips with the 5μg/mL threshold detected the progesterone after the LH surge just over 80% of the time, compared to ~60% detection frequency for the PDG rise with the 7μg/mL. Given these results, the newer lower threshold is more appropriate for clinical use. It should be noted that some older reproductive age women in the study might have lower post ovulation progesterone levels that may contribute to some of the negative tests.

The main limitation of our study was the small sample size with only 13 participants and 34 menstrual cycles of use. Having a larger number of participants that use the PDG test strips for 7 days or more is recommended. While the gold standard to identify ovulation is the use of ultrasound, as mentioned LH as measured by the CBEFM has a strong correlation with ovulation as demonstrated by established reference ranges (11).

Many women have started to use the new PDG test trips for various reasons; however, the main reason is to assist with achieving pregnancy. The PDG tests provide women with evidence that they have ovulated and have adequate progesterone levels and luteal phases that are long enough to support a pregnancy. Women who use FABMs to avoid pregnancy could also use the PDG test as a double check method for confirming the end of the fertile phase. The PDG tests could also be useful for women who have polycystic ovarian syndrome, have difficulty identifying their LH surge or peak mucus, are in postpartum amenorrhea and want to confirm their first ovulation, and women in perimenopause who want to confirm they are still ovulating. These applications have been evaluated before by others who have been able to measure PDG in the urine (6, 7), and require further follow-up with newer devices to identify whether these are viable applications for home PDG measurement. At present, the confirmation of ovulation in only 80% of the women in this study makes the practical value of the test still open to further investigation.

The urine PDG test is another way for women to test their hormones at home to determine if they have ovulated. Currently, there are PDG test strips in devolopment that can be interpreted by smart phone cameras rather than the subjectivity of the naked eye. Quantitative PDG tests are also being developed and should be available in the near future.

## Ethics Statement

This study was approved by the Marquette University Office of Research Compliance.

## Author Contributions

All authors listed have made a substantial, direct and intellectual contribution to the work, and approved it for publication.

### Conflict of Interest Statement

The authors declare that the research was conducted in the absence of any commercial or financial relationships that could be construed as a potential conflict of interest.
